# Hyper spectral resolution stimulated Raman spectroscopy with amplified fs pulse bursts

**DOI:** 10.1038/s41377-023-01367-0

**Published:** 2024-02-29

**Authors:** Hongtao Hu, Tobias Flöry, Vinzenz Stummer, Audrius Pugzlys, Markus Zeiler, Xinhua Xie, Aleksei Zheltikov, Andrius Baltuška

**Affiliations:** 1 Photonics Institute, Technische Universität Wien, Gußhausstraße 27-29, Vienna, A-1040 Austria; 2 SwissFEL, Paul Scherrer Institute, Villigen PSI, 5232 Switzerland; 3 Institute for Quantum Science and Engineering, Department of Physics and Astronomy, Texas A&M University, College Station, TX 77843 USA

## Abstract

We present a novel approach for Stimulated Raman Scattering (SRS) spectroscopy in which a hyper spectral resolution and high-speed spectral acquisition are achieved by employing amplified offset-phase controlled fs-pulse bursts. We investigate the method by solving the coupled non-linear Schrödinger equations and validate it by numerically characterizing SRS in molecular nitrogen as a model compound. The spectral resolution of the method is found to be determined by the inverse product of the number of pulses in the burst and the intraburst pulse separation. The SRS spectrum is obtained through a motion-free scanning of the offset phase that results in a sweep of the Raman-shift frequency. Due to high spectral resolution and fast motion-free scanning the technique is beneficial for a number SRS-based applications such as gas sensing and chemical analysis.

## Abstract (web summary)

We theoretically demonstrate a novel Stimulated Raman Scattering spectroscopy with hyper spectral resolution and high-speed spectral acquisition using offset-phase controlled fs-pulse bursts.

## Graphical abstract

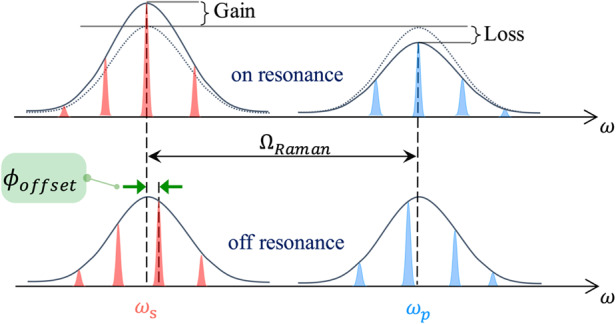

## Introduction

Stimulated Raman scattering plays a prominent role in the resonant nonlinear spectroscopy by addressing Raman-active (and thus optically-inactive) vibrational and rotational transitions, distinguishing itself from spontaneous Raman scattering^1^ by providing as reported 7 ~ 8 orders of magnitude stronger signals^2–4^. Since the first observation^5,6^, coherent Raman scattering has been widely applied in biological imaging^7–9^, environmental gas sensing^10–12^, materials characterization^13,14^, molecular dynamics tracking^15–17^, and other fields^18–23^.

Scanning the Raman-shift frequency, the molecular vibrational or rotational energy levels, is the most essential and crucial part of Stimulated Raman scattering applications. The choice of the driven laser fields to excite the molecules or materials is of vital importance since it fundamentally determines the spectral resolution and the way to achieve the Raman spectrum. Conventional techniques^8^ for high-spectral-resolution SRS spectroscopies involve the use of narrowband laser pulses where, to obtain an SRS spectrum, the center wavelength of at least one of the pulses is scanned step-by-step^24–30^. However, the step-by-step frequency scanning severely limits the speed of SRS spectral acquisition because of mechanical or thermal inertia^31^. On the other hand, parallel multiwavelength probing using a combination of a narrowband ps excitation and broadband fs probe corresponds to a suboptimal temporal pulse overlap and leads to many problems^32,33^. Above all, it requires suppression of a background signal originating from non-resonant four-wave mixing^34^.

In this work, we numerically demonstrate a novel approach to achieve the SRS spectrum by using the offset-phase controlled fs-pulse bursts, thus it can be called BSRS (burst-driven SRS). In BSRS, the Raman spectrum is accomplished by motion-free scanning that is simply and digitally changing the offset phase of the pulse bursts, which requires neither wavelength-detuning as in the long-pulse method nor precise dispersion management and delay scanning with movable parts as in the spectral focusing technique. The key strengths of the method proposed here are (i) the high spectral resolution, determined by the inverse duration of the pulse burst, and (ii) the rapid motion-free scan of the spectral mode across the targeted Raman resonance.

For the technique of BSRS, the numerical feasibility study precedes an actual experiment since we need theoretical calculations to provide quantitative estimations and answer several critical questions, thus demonstrating its practicality. In this method, we expect high spectral resolution due to the narrow spectral linewidths formed by the bursts to drive SRS. As the corresponding pump and Stokes linewidths directly derive from the burst duration, it is tempting to presuppose that the spectral resolution and spectral shape can be straightforwardly estimated, which needs to be confirmed by simulation. Also, in reality, the situation with a repetitive excitation with broadband two-color pulses is far more complex and unintuitive, thus requiring meticulous numerical validation. Moreover, uncontrolled accumulation of a background due to non-resonant four-wave mixing becomes a prominent issue as compared to conventional schemes that rely on a narrowband resonant coupling to a Raman transition. Therefore, a thorough description of SRS in the burst regime is required to answer several key questions: (1) Is this regime suitable for high-resolution SRS spectroscopy despite the large input pulse bandwidth? (2) How do the resonant and off-resonant spectral features evolve with time over the burst duration and what are the optimal burst parameters? (3) How does the SRS signal scale as a function of the number of pulses in burst? (4) Can the resonant SRS spectrum be reliably separated from the non-resonant four-wave-mixing background?

## Results

### Working principle

**Fig. 1:**
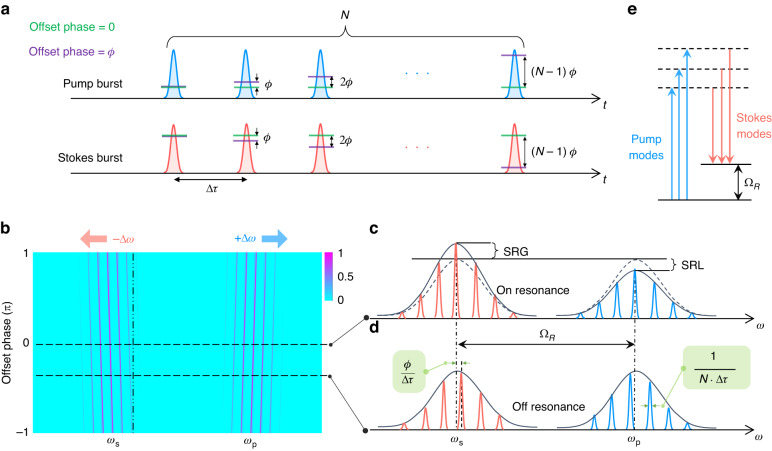
Working principle for Stimulated Raman Scattering driven by bursts. **a** Schematic diagram of the pump burst and the Stokes burst in the time domain with the offset phase equal to zero and $\phi$. *N*: number of pulses in burst. $\triangle \tau$: inter-pulse temporal separation. $\phi$: offset phase, the phase difference between two neighbouring pulses in burst. **b** The comb peaks belonging to the pump burst and the Stokes burst move in the opposite direction due to phase conjugation when the offset phase changes. **c** In the case of resonance, the comb peak frequency difference matches the Raman frequency, ${\Omega }_{R}$, leading to SRG (Stimulated Raman Gain) and SRL (Stimulated Raman Loss). The energy can flow from the pump burst to the Stokes burst via the vibrational excitation of a molecule. **d** For the off-resonance case, the Raman process is strongly suppressed and leads to minor changes in the burst pulse energies depending on the detune. **e** Simultaneous stimulated Raman on-resonance is achieved by multiple pumps and Stokes comb frequencies

The main technical prerequisite for implementing the method in Fig. 1 has been realized in our group^35^ based on a programmable generation of amplified fs pulse bursts in a regenerative amplifier (RA) operated in the Vernier mode relative to the cavity length of the master oscillator (MO). As experimentally shown in ref. ^35^, spectral interference of the pulses in the generated bursts leads to a comb-like structure with a $$1{THz}\simeq 33.3$$$$c{m}^{-1}$$ intermodal distance when the detuning between the RA and MO roundtrips $$\triangle \tau =\left|{L}_{{RA}}-{L}_{{MO}}\right|/c=1{ps}$$. Please note, the $$\triangle \tau $$ can be adjusted by changing the cavity length difference between the RA and MO. The THz-spaced pseudo-modes have the spectral width determined by the inverse duration of the burst, $${\left(\triangle \tau \cdot N\right)}^{-1}$$, are filled with a very dense, MHz-interval-frequency comb structure^36^ that is irrelevant to our study. The peak frequencies of the pseudo-modes, corresponding to constructive spectral interference, are programmed, alongside the individual amplitudes of the pulses forming the burst, by use of an AOM placed between the MO and RA. In practice, the offset phase can be controlled by an AOM working at the frequency of $$300 \sim 400$$ $${MHz}$$. Considering the MO working frequency is around 100 $${MHz}$$, AOM actually is able to achieve the intensity and phase controlling of each pulse one by one within a burst.

We note that the aspect of chirp management becomes irrelevant for the spectral narrowing achieved in this case purely through interference. Correspondingly, a white-light-seeded OPA driven by such a pulse burst will behave exactly like an OPA driven by a single isolated fs laser. As a result, an independent signal-idler pulse pair will originate from each laser pulse in the burst and similar pseudo-combs will arise under the respective spectral envelopes (Fig. 1) through the spectral interference of signal and idler pulse bursts. We further note that due to phase conjugation in a properly designed OPA^37^, the phase control of the fundamental laser burst will cause spectral modes to shift in the opposite directions for the signal and idler pseudo-combs. Therefore, as shown in Fig. 1c, d, resonant SRS conditions can be simultaneously fulfilled or missed by multiple pseudo-modes depending on the input phase of the pulses loaded into the RA. This is the motion-free scanning to achieve the Raman spectrum.

In this and the following paragraph, we explain in more detail the phase-conjugation and the spectral range, respectively. The anti-phase (or conjugate-phase) of the pump and Stokes beams is chosen in this work for the sake of intuitiveness and the symmetry of the formalism. In an actual configuration of the OPA, depending on the choice of the pair of output waves or their optical harmonics, the scaling factors connecting the magnitude of frequency mode shift to the input phase shift in the AOM can be different from those assumed in our model. Various configurations, enabling spectral acquisition, are possible as long as the frequency sweep rates as a function of the phase offset on the fundamental-frequency burst are not identical for the pump and the Stokes bursts. The simplest scenarios involve signal/fundamental + idler from a fundamental-pumped and a fundamental-white-light OPA (Configuration A in Table I of ref. ^37^), whereby the idler and its harmonics are rendered with a constant phase. Under such conditions, the phase sweep would shift the pump-burst frequency modes while the Stokes-burst modes would remain steady.

The free spectral range, FSR, of the optical frequency sweep is determined by the interpulse spacing in the pulse burst, $\frac{33.3{c}{m}^{-1}}{\triangle \tau [{ps}]}$. In this numerical feasibility study, we assume a full-width at half-maximum (fwhm) 100-${fs}$ pulse duration, which supports burst frequencies above $1{THz}$ ($\triangle \tau =1{ps}$, FSR = $33.3{c}{m}^{-1}$) without significant temporal overlap between the neighboring pulses in the pulse train. Correspondingly, broadband laser amplifiers and OPAs delivering shorter pulses durations, such as conventional fs 800-${nm}$ Ti:sapphire systems, have the potential to cover well over $333{c}{m}^{-1}$ ($\triangle \tau$ is as small as 0.1 ps) in a single frequency sweep across an FSR between neighboring spectral modes. Please note, that by reducing $\triangle \tau$ alone, one can achieve a broader spectral scanning range. Nevertheless, the spectral resolution will be lower due to the decreased total burst duration ($N\cdot \triangle \tau$). To simultaneously achieve a broader spectral scanning range and maintain the high spectral resolution, one need to reduce $\triangle \tau$ and increase $N$, keeping the total burst duration as constant. On the other hand, the maximum value of $\triangle \tau$ normally should not be larger than a few tens of ${ps}$ to maintain a free spectral range wider than a few $c{m}^{-1}$. To interrogate a different vibrational transition, the detuning between the center frequencies of the pump and Stokes bursts, with a precision within an FSR, is set to a new approximate value by tuning the OPA in a conventional way. Then the motion-free phase scan is repeated. In short, in the proposed technique, the center wavelengths are roughly set and then frequency modes under a fixed spectral envelope are scanned by a phase sweep.

**Fig. 2:**
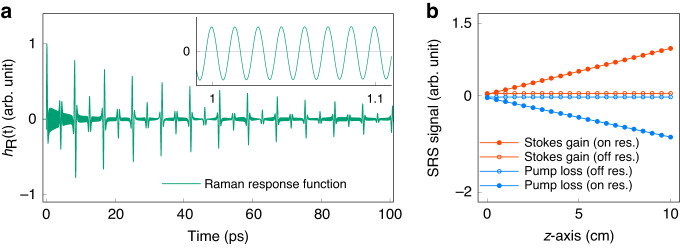
Raman response function of molecular nitrogen and the SRS signal versus the propagating distance. **a** Raman response function of molecular nitrogen including the rovibrational Q-branch transitions and the rotational transitions. The insert shows the fast oscillation from $1{ps}$ to $1.1{ps}$. **b** SRS signal (SRG and SRL) versus the propagating distance along the z-axis. The points with solid circles represent the resonant case and the points with open circles for the non-resonant case

In the following analysis, we choose molecular nitrogen (*N*_2_) as an example, since it is sufficient to demonstrate the proposed method, and the complexity of samples is not expected to significantly impact the results in this work. Figure 2a shows the Raman response function of *N*_2_ over time. The high-frequency oscillations shown in the insert figure are from the rovibrational Q-branch transitions around $2330{c}{m}^{-1}$. The full revival with a period of $8.4{ps}$ originates the rotational transitions. The Raman response function presented in Fig. 2a is perfectly consistent with recent experimental measurements with very high sensitivity^38^. The detailed parameters of the Raman response function for molecular nitrogen and oxygen could be found in refs. ^39–41^. Figure 2b shows the variation of laser intensity for Stokes burst (red) and pump burst (blue) as a function of the propagating distance in space. In the simulation, the inter-pulse temporal separation ($\triangle \tau$) is $1{ps}$, and the number of pulses ($N$) is 100. As illustrated in Fig. 1, by changing the offset phase, one can tune the SRS between on-resonance (solid circles) and off-resonance (open circles). For the resonant offset phases, the pulse energy can sufficiently flow from the pump burst to the Stokes burst, resulting in their loss and gain respectively. The linear growth of intensity over propagation distance is consistent with the formula deduced in^21^ in the weak signal limit.

### Raman shift scanning

**Fig. 3:**
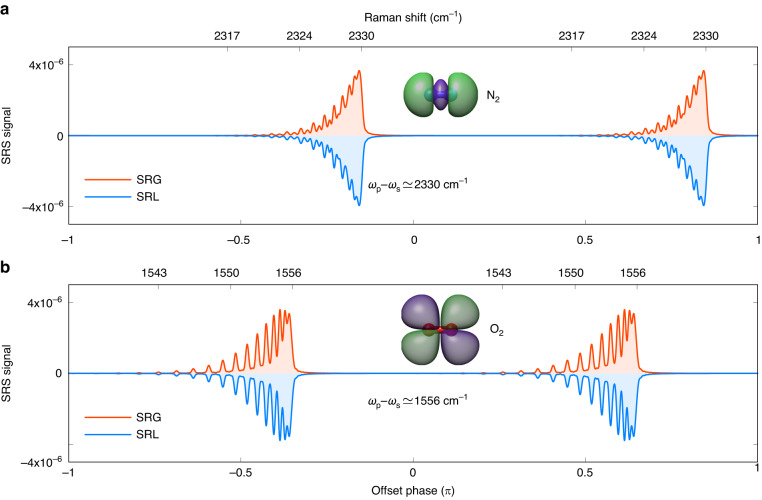
Burst-driven stimulated Raman shift spectra of molecular nitrogen and molecular oxygen. Raman shift spectrum of burst-driven SRG (red curve) and SRL (blue curve) around $2330{c}{m}^{-1}$ in *N*_2_ (**a**) and around $1556{c}{m}^{-1}$ in *O*_2_ (**b**) by changing the offset phase from $-\pi$ to $\pi$. The upper axis is the Raman shift energy in $c{m}^{-1}$ corresponding to the offset phase. In those simulations, $N=100,\triangle \tau =1{ps},{I}_{s}/{I}_{p}=0.8$

Figure 3 shows the calculated Raman shift spectrum of Q-branch ($\triangle J=0$) associated with the vibrational transition $\upsilon =0\to \upsilon =1$ of molecular nitrogen (a) and molecular oxygen (b) in their ground states ${X}^{1}{\Sigma }_{g}^{+}$ and ${X}^{3}{\Sigma }_{g}^{-}$ respectively. With the present spectral resolution, ${\left(N\cdot \triangle \tau \right)}^{-1}=0.33{c}{m}^{-1}$, the *J* state ratio of both molecules is clearly shown in the Raman shift spectra. *N*_*2*_ has a nuclear spin quantum number of 1, so its even-*J* to odd-*J* ratio is 2:1. The nuclear spin quantum number for *O*_2_ is 0, and its even-*J* is forbidden. The simulations shown in Fig. 3 are nicely agreed with previous experimental results^40,41^. The periodic repetition of every $\pi$ is also revealed in Fig. 3, coming from the periodic property of the offset phase, which is a unique feature of BSRS and indicates an easy phase-to-frequency calibration procedure in practice. The signal yield ratio between SRG and SRL can also help the calibration procedure and could be estimated by $\frac{{SRG}}{{SRL}}=\frac{{I}_{p}{\omega }_{s}}{{I}_{s}{\omega }_{p}}$, which is $\simeq 1$ under current considerations.

It needs to be emphasized that although in Fig. 3 the spectral scanning range is only $33{c}{m}^{-1}$, this method is not restricted in terms of spectral coverage but only in terms of an FSR, over which a motion-free frequency sweep can be performed. Broad spectral range can be attained by tuning the OPA, which acts in a way as a bandpass filter. Our method does not fully eliminate the need for frequency tuning, but it dramatically speeds up spectral acquisition by avoiding a slow consecutive point-by-point center wavelength scan. Correspondingly, addressing multiple spectral ranges of interest in other applications, like the biological imaging, is also straightforward.

## Discussion

### Spectral resolution

**Fig. 4:**
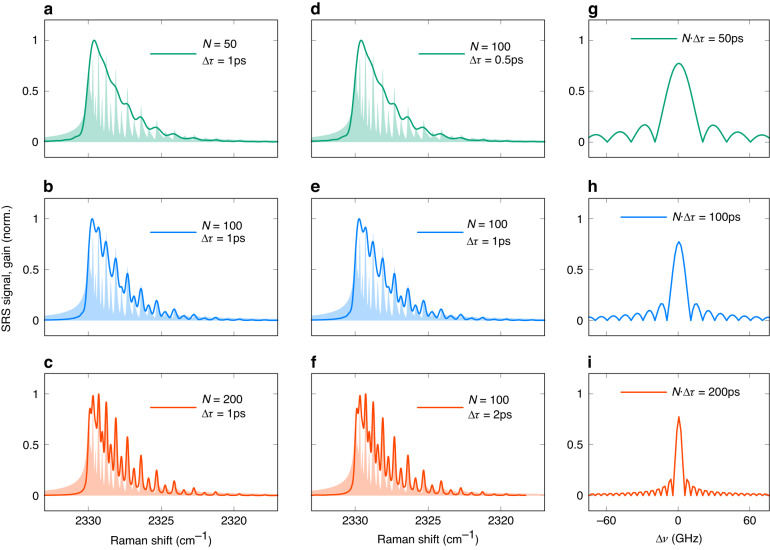
Spectral resolution dependence on the number and the inter-pulse temporal separation of pulses in a burst. Normalized Raman shift spectrum for (**a**, **d**) $N\cdot \triangle \tau =50{ps}$, (**b**, **e**) $N\cdot \triangle \tau =100{ps}$ and (**c**, **f**) $N\cdot \triangle \tau =200{ps}$. **a**–**c** Changing the number of pulses, keeping the inter-pulse temporal separation unchanged. **d**–**f** Changing the inter-pulse temporal separation and keeping the number of pulses unchanged. **g**–**i** Width of individual peaks in the comb for different values of $N\cdot \triangle \tau$: (**g**) $N\cdot \triangle \tau =50{ps}$, (**h**) $N\cdot \triangle \tau =\,100{ps}$, and (**i**) $N\cdot \triangle \tau =\,200{ps}$. The filled areas in (**a**–**f**) present the Raman spectrum from the direct Fourier transform of the Raman response function

The high spectral resolution is desired to separate the species if their Raman shift spectra distributions are close to or overlap with each other. Figure 4a–f shows that a higher spectral resolution is achieved by increasing either $N$ or $\triangle \tau$ or both. It confirms that the spectral resolution in BSRS is given by the reciprocal of the product of the number of pulses and the inter-pulse temporal separation, ${\left(N\cdot \triangle \tau \right)}^{-1}$. It can be seen that as $N\cdot \triangle \tau$ increases from $50{ps}$ to $200{ps}$, the spectral peaks become narrower, and the distinction between even *J* and odd *J* becomes more pronounced. The corresponding spectral resolution of (**a**, **d**) is $0.66{c}{m}^{-1}$, (**b**, **e**) is $0.33{c}{m}^{-1}$, and (**c**, **f**) is $0.17{c}{m}^{-1}$. Figure 4g–i shows the spectral width of the individual peak in comb getting narrower and narrower when $N\cdot \triangle \tau$ increases from $50{ps}$ to $200{ps}$. Figure 4c and f also confirms that the spectral resolution is always $0.17{c}{m}^{-1}$ for any configuration of $N\cdot \triangle \tau =200{ps}$, like $N=200$; $\triangle \tau =1{ps}$ or $N=100$; $\triangle \tau =2{ps}$ or $N=50$; $\triangle \tau =4{ps}$, etc.

A single-pulse driven field with a fwhm of $200{ps}$ can also achieve such high spectral resolution, however, its peak intensity is limited by the ps laser technology. On the one hand, the burst has a higher laser intensity than the single pulse field when the total pulse energy is the same. For example, the laser intensity of a burst with *N* = 200 and fwhm = 100 *fs* is ten times higher than that of a signal pulse with fwhm = $200{ps}$ when they have the same total laser pulse energy. On the other hand, the chirped pulse amplification technique allows more energy to be put into fs bursts. SRS is a non-linear process, and its signal is directly determined by laser intensity with the relation of $\triangle {I}_{s,p}/{I}_{s,p}\propto {Im}({\chi }_{R}^{(3)}){I}_{p,s}L$, where ${\chi }_{R}^{(3)}$ represents the third-order nonlinear susceptibility, and $L$ indicates the interaction length. SRS driven by a higher intensity field thus has higher detection sensitivity, which can detect molecules of interest with lower counts in the sample and requires a shorter sample thickness to obtain the same signal yield. It is important to note that the laser intensity must be kept below the damage threshold of the target sample.

### Resonant mechanism

**Fig. 5:**
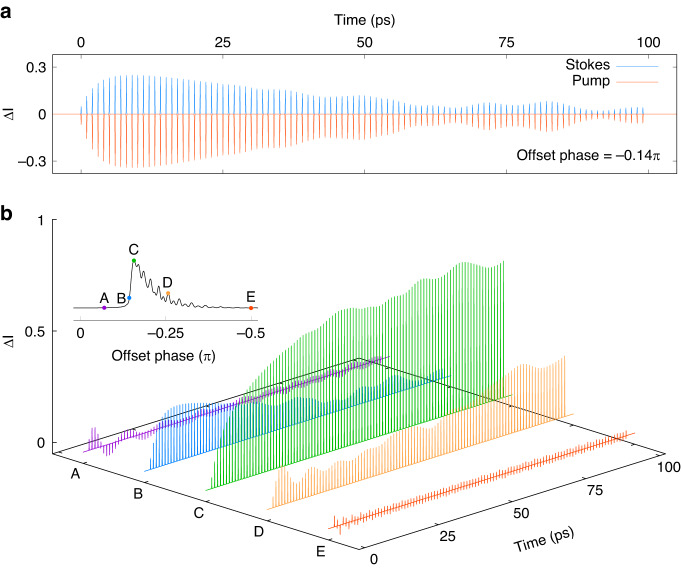
Signal accumulation over time for different offset phases. Intensity difference between the driven burst before and after the Stimulated Raman process, $\triangle {I}_{s,p}\left(t\right)={\left|{A}_{s,p}\left(z,t\right)\right|}^{2}-{\left|{A}_{s,p}\left(z=0,t\right)\right|}^{2}$. **a** $\triangle {I}_{s}(t)$ and $\triangle {I}_{p}(t)$ as a function of time when the offset phase is -$0.14\pi$. **b** $\triangle {I}_{s}(t)$ for different offset phases. The offset phases corresponding to the points are shown in the insert figure, their values are *A* = −0.07 $\pi$, *B* = −0.14 $\pi$, *C* = −0.16 $\pi$, *D* = −0.26 $\pi$, *E* = −0.5 $\pi$

Figure 5 intends to explain how the signal buildup in the time domain by showing the change of the signal after each pulse in the burst ($N=100,\triangle \tau =1{ps}$). Figure 5a depicts $\triangle I(t)$ for the Stoke burst (red curve) and the pump burst (blue curve), which shows their coupling obviously. Due to the conservation of photon numbers, the equation $\frac{\triangle {I}_{s}(t)}{{\omega }_{s}}+\frac{\triangle {I}_{p}(t)}{{\omega }_{p}}=0$ holds all the time. Thus, $\triangle {I}_{s}(t)/\triangle {I}_{p}(t)$ shown in Fig. 5a is around 0.8 for the whole time domain.

As described in ref. ^42^, the initial phase of the vibrational dipole (or the Raman oscillation) induced by the driving pump and Stokes fields is determined by their phase difference. If the offset phases of the driving laser fields satisfy a condition that vibrational dipoles induced by two adjacent pulses interfere constructively, a strong signal buildup takes place. The condition is that the difference between the model peaks of the pump burst and Stokes burst equals to the molecular Raman frequency, as shown in Fig. 1c, d. Figure 5b shows that the gain and loss of driven laser fields happen in every pulse, which is reasonable since each pair of fs-pulses in Stoke and pump bursts can induce the SRS process. However, by integrating $\triangle I(t)$ over time, it is found the net gain and loss, the accumulation of signal, only exist for the resonant offset phases, like Point B, C and D. For the non-resonant offset phases, like Point A and E, the negative and positive parts completely cancel out, leading to zero Raman signal. The range and value of the net gain are mainly determined by the value of the offset phase. For example, from Point A to Point C, the range of net gain grows from a few ${ps}$ to $100{ps}$, and a more than tenfold increase in value. Point C has the strongest resonance, every pulse in the burst constructively contributes to the net gain at a high level of value. At this point, the Raman molecular vibration induced by each pair of pumps and Stokes pulse can constructively superimpose and thus give a strong net gain all over the whole burst.

### Signal yield

**Fig. 6:**
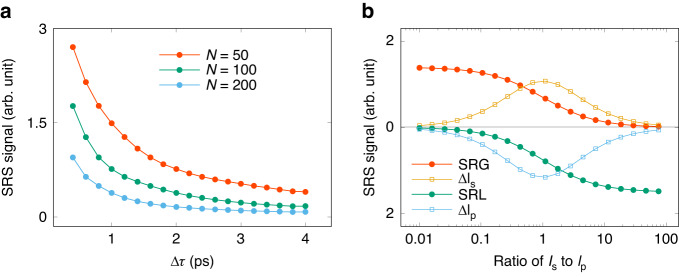
Dependence of Raman signal yield on driving laser burst parameters with a fixed total laser pulse energy. **a** number of pulses in burst and the inter-pulse temporal separation. (**b**) laser intensity ratio of Stokes burst to pump burst. The total pulse energy is fixed for those results

Figure 6 presents the effect of the driven laser parameters, including the number of pulses *N*, the inter-pulse temporal separation $\triangle \tau$, and the intensity ratio ${I}_{s}/{I}_{p}$, on the stimulated Raman signal. The total laser pulse energies are the same for all simulations in Fig. 6. In Fig. 6a, the blue line-points (*N* = 200) are below the red line-points (*N* = 50) which indicates the signal strength decreases as *N* increases. The reason is that a larger *N* leads to a smaller energy per pulse in the burst and a lower peak laser intensity resulting in a decrease in the signal yield. We also see increasing $\triangle \tau$ leads to a decrease in yield for every value of *N*. The decrease of fast oscillations around $0 \sim 10{ps}$ of the Raman response function in Fig. 2a give an explanation: as $\triangle \tau$ increases, the pulse-to-pulse superposition becomes less effective which reduces the efficiency of SRS. A larger value of $N\cdot \triangle \tau$ means higher spectral resolution and lower SRS signal yield, thus Fig. 6a tells us that when the total laser pulse energy is fixed, we can get either a high spectral resolution with relatively lower SRS signal yield, or a high SRS signal yield with relatively lower spectral resolution. As discussed, $\triangle \tau$ can affect the Raman scanning range which equals to $\frac{33.3{c}{m}^{-1}}{\triangle \tau [{ps}]}$, so $\triangle \tau =4{ps}$ corresponds to a scanning range of $7{c}{m}^{-1}$. Therefore, we only calculate $\triangle \tau$ scanning up to $4{ps}$ to cover the rovibrational spectrum of *N*_2_ of interest.

The effect of laser intensity ratio to the signal yield is depicted in Fig. 6b, in which $\triangle \tau =1{ps}$ and *N* = 100. We observe that ${SRG}=\triangle {I}_{s}/{I}_{s}$ is higher when ${I}_{p}\gg {I}_{s}$, while ${SRL}=\triangle {I}_{p}/{I}_{p}$ prefers ${I}_{p}\ll {I}_{s}$, which is consisted with ref. ^21^. The open square points show the absolute changes of $\triangle {I}_{s}$ and $\triangle {I}_{p}$. The peak position for them locate at ${I}_{s}={I}_{p}$. A previous research^43^ showed that to achieve a high signal-to-noise ratio, the laser intensity ratio should be set as ${I}_{p}/{I}_{s}=2$ for SRG and ${I}_{s}/{I}_{p}=2$ for SRL.

**Fig. 7:**
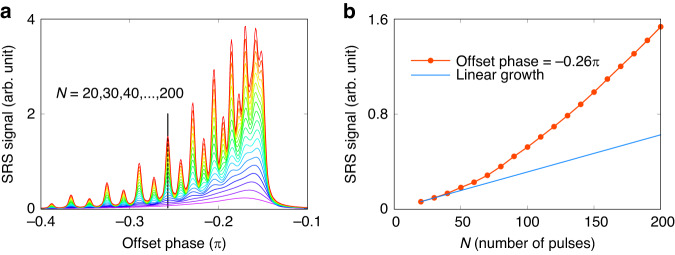
Raman signal yield versus number of pulses (*N*) for a fixed laser intensity. **a** the corresponding N from bottom to top is $20,30,\ldots ,200$. **b** is the data of the offset phase equals to −0.26 *π*, indicated by the vertical black line in (**a**). $\triangle \tau$ is $1{ps}$ in those simulations

In addition to the signal yields for a fixed total laser pulse energy of bursts, as shown in Fig. 6, we have also investigated the signal yield as a function *N* when the laser intensity is fixed, which means the total laser pulse energy of bursts increases as *N* increasing, to reveal the coherent growth laws. Figure 7a shows the Raman shift spectra for *N* increasing from 20 to 200. It can be seen that the resolution increases as *N* increases. For *N* = 20, the Raman shift spectrum (bottom purple curve) has a broad peak that can not separate the resonance or non-resonance narrow peaks. For *N* = 200, the Raman shift spectrum (top red curve) clearly shows the rovibrational structures of the nitrogen, and the odd-*J* and even-*J* can be distinguished easily.

Another feature is that as *N* increases, the signal yield becomes larger, especially at the resonant frequency of the nitrogen. The red dotted line in Fig. 7b shows the signal yield growth at offset phase = −$0.26\pi$ indicated by the vertical black line in Fig. 7a. The reason for faster than linear growth (blue line) is the offset phase at −$0.26\pi$ corresponds to the resonance frequency of the *N*_2_, thus the signal yield grows coherently when increasing *N* due to constructive interference of the signals from each pulse pairs in the bursts. In Eq. (2), the sum part ${\sum }_{m=0}^{N-1}\exp ({im}\alpha )$ is equal to $\frac{\sin \left(N\alpha /2\right)}{\sin \left(\alpha /2\right)}{e}^{-i\left(N-1\right)\alpha /2}$, where $\alpha$ is set as $\Omega \cdot \triangle \tau -2\phi$. In the experiment, the measured signal is the laser intensity, so we need to investigate the signal growing properties with *N* by the square of the sum part, which is ${|{\sum }_{m=0}^{N-1}{{\rm{e}}}^{{im}{\alpha }}|}^{2}=\frac{{\sin }^{2}\left(N\alpha /2\right)}{{\sin }^{2}\left(\alpha /2\right)}$. And when $\alpha \to 0$, we have the pure quadratic growth with *N* as ${\mathrm{lim}}_{\alpha \to 0}\frac{{\sin }^{2}\left(N\alpha /2\right)}{{\sin }^{2}\left(\alpha /2\right)}={N}^{2}$. Such a condition can be achieved when the Raman response function consists of only one frequency without any relaxation time, which corresponds to a Delta function in the frequency domain. However, the real Raman response function contains different frequencies with a certain width, the Raman signal includes also the contribution from the off-resonant frequency components, which grows lower than the quadratic. Therefore, the signal growth over *N* shown in Fig. 7b exhibits rather a mixture of quadratic and linear growth. The nonlinear growth of the signal yield over *N* indicates that a higher signal-to-noise ratio can be achieved with the BSRS as compared to those methods with single pulses.

The numerical results discussed above have answered the four questions proposed in the beginning. Specifically, Fig. 4 confirms the relation between the spectral resolution and the burst duration; Fig. 5 reveals the difference of the signal accumulation for the on- and off- resonant cases, and Fig. 6 gives the signal yield dependence on the burst parameters; Fig. 7 investigates the quadratic growth rules ensuring that the resonant SRS signal can be separated from the non-resonant background. Therefore, this numerical study undoubtedly demonstrates the feasibility of the BSRS technique, and provides a solid theoretical foundation for any further experimental implementations.

### Challenge and applications

To realize this unique method, several possible technical challenges in experiment need to be considered. A few years ago, a burst with 6 pulses has already been generated in our lab^35^, while to achieve a high spectral resolution *N* should be increased to 100. Since the first pulse and the last pulse in the burst have different round-trip numbers in the optical amplifier, the dispersion effects may need consideration. Recently long ${fs}$ pulse ${THz}$ burst (*N* = 40) with a time jitter *<* 0.01% of the ${ps}$ interpulse interval have been experimentally produced^44^. The associated frequency jitter of spectral modes is on a ${MHz}$ scale and presents no obstacle for resolving 10-GHz-spaced rovibrational modes considered in our numerical simulation. Secondly, the choice of ${f}_{R}$. ${f}_{R}$ refers to the magnitude fraction between the nonresonant background signal to the stimulated Raman signal. In our simulation, ${f}_{R}$ was set as 0.95 based on the fact that the resonant signal yield is a mixture of quadratic growth and linear growth, which is much higher than the frequency-irrelative non-resonant signals. The exact value of ${f}_{R}$ is related to the signal-to-noise ratio and can be determined by fitting the measured experimental results.

By overcoming those technical issues, BSRS can become a powerful and robust tool with very high spectral resolution and high-speed spectral acquisition. Due to high spectral resolution and fast scanning the technique of BSRS is beneficial for gas sensing which includes: (a) chemical analysis for identification of the gas compounds in mixtures and analyze the molecular structure changes; (b) environmental detection, to accurately detect the low concentrations of gases that may cause air pollution; (c) isotope analysis, to label the isotopes and characterize their percentage. This highspectral resolution technique will also benefit the development of tracking molecular dynamics^15–17^, in which an acting pump is used to initial the motion of vibration wave-packet and followed by the time-delayed Raman pump and Stokes pulses, providing a time-resolved SRS. On the one hand, the motion-free spectral scanning can potentially reduce the errors introduced by mechanical jitter in the experiments. On the other hand, the nonlinear growth of the signal as a function of the number of pulses in the burst, as shown in Fig. 7b, leads to a high signal-to-noise ratio.

Except for the gas targets, the BSRS technique also holds promising applications in biological imaging due to its exceptional spectral resolution and free-motion scanning capability. While the spectral resolution of existing techniques typically ranges from $3 \sim 5{c}{m}^{-1}$ for the picosecond pulse-driven methods^25,45^ to 10~30*cm*^-1^ for the femtosecond pulse-driven methods^21,31,46,47^. Our method can achieve a spectral resolution of $0.17{c}{m}^{-1}$ when $N\cdot \triangle \tau =200{ps}$. As previously discussed, the motion-free spectral scanning and the nonlinear growth of the signal are expected to enhance the signal-to-noise ratios. Additionally, the short spectral detuning time in this method, which is $10\mu s$ when the OPA operates at a repetition rate of $100{kHz}$, enables the high-speed Raman spectrum acquisition. That is another advantage of this method over many methods in which the achievement of the frequency detuning is challenging.

In conclusion, we demonstrate numerically a novel approach to achieve the Stimulated Raman spectrum with high spectral resolution by using offset-phase controlled fs-pulse bursts. This work offers innovations in both fundamental and technical aspects. From the fundamental point of view, we successfully demonstrated the feasibility of this method in which a narrowband signal can be retrieved by applying a set of broadband pulses and quantitatively investigating how the growth of the SRS signal depends on the number of pulses in burst. Technically, we introduced a motion-free scanning method to detect SRS spectrum possessing many advantages, like hyperspectral resolution, high spectral sweep speed and high detection sensitivity. There are many attractive applications of this method, like gas sensing, chemical analysis, environmental pollution detection, isotope characterization, and molecular dynamics tracking.

## Materials and methods

Using molecular nitrogen as a model system, we present systematic numerical simulations, based on the coupled nonlinear Schrödinger equations^48^ shown in Eq. (1). A detailed procedure for the derivation of the coupled nonlinear Schrödinger equations can be found in the literature^49,50^.

1 $$\begin{array}{c}\frac{\partial {A}_{p}}{\partial z}=i{\gamma }_{p}\left(1-{f}_{R}\right)\left({\left|{A}_{p}\right|}^{2}{A}_{p}+2{\left|{A}_{s}\right|}^{2}{A}_{p}\right)+{R}_{p}\left(z,t\right)\\ \frac{\partial {A}_{s}}{\partial z}=i{\gamma }_{s}\left(1-{f}_{R}\right)\left({\left|{A}_{s}\right|}^{2}{A}_{s}+2{\left|{A}_{p}\right|}^{2}{A}_{s}\right)+{R}_{s}(z,t)\end{array}$$

In the equations, ${A}_{p}$ and ${A}_{s}$ are the slowly varying envelopes associated with the pump and Stokes pulse bursts, ${\gamma }_{p,s}$ is the nonlinear parameters, ${f}_{R}$ represents the fractional contribution of the resonant Raman response to nonlinear polarization^21,48,51,52^. ${R}_{p,s}(z,t)$ shown in Eq. (2) is the Raman contribution term, which is a function of the frequency detuning ($\Omega$) and Raman response function [${h}_{R}(t)$]. The Raman response function of molecular nitrogen^39,40^ is depicted in Fig. 2a. Equations (1) and (2) are valid as long as the slowly varying envelop approximation is true, a condition that holds for pulses with a pulse duration > 10 fs in the visible and near-infrared regions^49,50^

2 $$\begin{array}{ll}{R}_{p,s}\left(z,t\right)=i{\gamma }_{p,s}{f}_{R}{A}_{p,s}{\int }_{-{\infty }}^{t}d{t}^{{\prime} }{h}_{R}(t-t^{\prime} )\left({\left|{A}_{p,s}(z,t^{\prime} )\right|}^{2}+{\left|{A}_{s,p}(z,t^{\prime} )\right|}^{2}\right)\\\qquad\qquad+\,i{\gamma }_{p,s}{f}_{R}{A}_{s,p}{\int }_{-{\infty }}^{t}d{t}^{{\prime} }{h}_{R}\left(t-{t}^{{\prime} }\right){A}_{p,s}\left(z,{t}^{{\prime} }\right){A}_{s,p}^{* }(z,t^{\prime} )\exp\\\qquad\qquad (\pm i\Omega (t-t^{\prime} ))\mathop{\sum }\limits_{m=0}^{N}\exp ({im}(\Omega \cdot \triangle {\rm{\tau }}-2{\rm{\phi }}))\end{array}$$

The electric field of the pump and Stokes pulse bursts, ${E}_{p,s}={A}_{p,s}\cos ({\omega }_{p,s}t+{\Phi }_{p,s})$, are given by Eq. (3), with the laser intensity (${I}_{p,s}$) around 10^11~12^ $W/c{m}^{2}$, the pulse duration (${\upsilon }_{p,s}$) of individual pulses in burst equals to 100 fs. ${\omega }_{p}$ and ${\omega }_{s}$ are the respective central carrier frequency of the broadband individual pulse in the pump burst and Stokes burst, which is approximately detuned to the Raman shift frequency, ${\Omega }_{{\rm{R}}}\approx {\omega }_{p}-{\omega }_{s}$. The offset phase ($\phi$) is assumed to be constant over one pulse in the burst^35^. The frequency detuning is ${\omega }_{p}-{\omega }_{s}$, denoted by $\Omega$. ${\lambda }_{p}=919{nm}$ and ${\lambda }_{s}=1170{nm}$ were chosen such that their angular frequency difference locates in the range of the Raman shift ($2330{c}{m}^{-1}$) of molecular nitrogen and the sum of the photon energies of a 919-*nm* and a 1170-*nm* photons corresponds to a pump photon energy with the 515 nm wavelength, i.e. we are referring to an OPA pumped by a frequency-doubled 1030*nm* Yb-doped laser^53^. Additional to molecular nitrogen, we also performed simulations with molecular oxygen using the same model. For molecular oxygen, the Raman shift is around 1556*cm*^-1^, and the wavelengths were set as ${\lambda }_{p}=965{nm}$ and ${\lambda }_{s}=1135{nm}$. Due to constructive conditions between pulses in the burst, the offset phase ($\phi$) and the inter-pulse temporal separation ($\triangle \tau$) are relative to the frequency detuning naturally. The Stimulated Raman Gain (${SRG}$) and Stimulated Raman Loss (${SRL}$) are given by ${SRG}={g}_{s}=\frac{{\triangle I}_{s}}{{I}_{s}}$ and ${SRL}={l}_{p}=\frac{{\triangle I}_{p}}{{I}_{p}}$, respectively.

3 $$\begin{array}{c}{E}_{p}=\mathop{\sum }\limits_{m=0}^{N-1}\sqrt{{I}_{p}}\exp \left[-\frac{{\left(t-m\Delta \tau \right)}^{2}}{{\left({\upsilon }_{p}/2\right)}^{2}}\mathrm{ln}2\right]\cos ({\omega }_{p}(t-m\Delta \tau )-m\phi )\\ {E}_{s}=\mathop{\sum }\limits_{m=0}^{N-1}\sqrt{{I}_{s}}\exp \left[-\frac{{\left(t-m\Delta \tau \right)}^{2}}{{\left({\upsilon }_{s}/2\right)}^{2}}\mathrm{ln}2\right]\cos ({\omega }_{s}\left(t-m\Delta \tau \right)+m\phi )\end{array}$$

We know from the working principle that the frequency detuning can be realized through simply changing the offset phase ($\phi$). Based on the interference conditions of pulses in burst, the frequency detuning can be estimated by the formula of ${\Omega }_{{detuning}}=\left(\frac{{M}_{p}\cdot 2\pi }{\Delta \tau }+\frac{\phi }{\Delta \tau }\right)-\left(\frac{{M}_{s}\cdot 2\pi }{\Delta \tau }-\frac{\phi }{\Delta \tau }\right)$, where ${M}_{p,s}$ is the nearest integer of $\frac{{\omega }_{p,s}\cdot \triangle \tau }{2\pi }$. This formula indicates that the scanning range is determined by both the central frequency difference (${\omega }_{p}-{\omega }_{s}$) and the inter-pulse temporal separation ($\triangle \tau$). As discussed in the Working principle part, ${\omega }_{p}-{\omega }_{s}$ can be used to determine the general scanning range, and $\triangle \tau$ defines the fine spectral scanning range.

It is worth to mention that there is a possibility that two Raman frequencies (${\Omega }_{1}$ and ${\Omega }_{2}$) originating from different vibrational or rotational bands appear on the same position along the offset phase axis. This occurs because a single offset phase can correspond to multiple Raman detuning frequencies as ${\Omega }_{{detuning}}=\frac{({M}_{p}-{M}_{s}+m)\cdot 2\pi }{\Delta \tau }+\frac{2\phi }{\Delta \tau }$, where $m=\cdots ,-2,-\mathrm{1,0,1,2},\cdots$. However, this spectral overlap issue can be resolved in an actual experiment by repeating the scan with two different FSR. Initially, we can perform a broad-range scan, like $\triangle \tau =100{fs}$, to gain an overview of the broadrange spectrum of the system, enabling us to identify Raman frequencies, e.g. ${\Omega }_{1}$ and ${\Omega }_{2}$, which are possibly overlapped in a narrow scanning spectral range. For the broadrange scanning, since the ratio of $\triangle \tau$ to the pulse duration (suppose ${\upsilon }_{p,s}\approx 40{fs}$) is as low as 2.5, ${\Omega }_{{detuning}}$ and $\phi$ maintain a nearly one-to-one correspondence. Subsequently, we can execute a high-spectral-resolution scan ($\triangle \tau$ is around ${ps}$) in the region of interest. To avoid the overlapping of two Raman frequencies along offset phase scan, we just need to make sure that $\triangle \Omega ={\Omega }_{1}-{\Omega }_{2}$ is not an integer multiple of $2\pi /\triangle \tau$.
